# Management of post-operative anaemia in patients undergoing surgery for colorectal cancer: a qualitative focus group-based study

**DOI:** 10.1007/s00384-024-04794-6

**Published:** 2025-01-15

**Authors:** Samuel Lawday, Susannah Williams, Elizabeth James, Emma L. Court, Fiona Carter, Francesca Rushton, Cat Dampier, E. J. O’Malley, M. Barrington, Rob Bethune, Nader Francis

**Affiliations:** 1 Centre for Surgical Research, Population Health Science, University of Bristol, Bristol, UK; 2 Somerset NHS Foundation Trust, Yeovil, UK; 3 Royal Devon University NHS Foundation Trust, Exeter, UK; 4 University of Exeter, Exeter, UK; 5 Department of Surgical Biotechnology, University College London, London, UK; 6 The Griffin Institute, Northwick Park Hospital London Northwick Park and St Mark’s Hospital, Y Block, Watford Rd, Harrow, HA1 3UJ UK

**Keywords:** Postoperative, Anaemia, Colorectal, Qualitative, Focus group

## Abstract

**Background:**

Optimal management of anaemia following surgery for colorectal cancer remains unclear. Peri-operative anaemia is common in patients undergoing resectional surgery for colorectal cancer. A significant amount of research has been conducted into the management of pre-operative anaemia; however, little work has investigated post-operative anaemia. We intended to investigate the facilitators of and barriers against the standardised correction of post-operative anaemia. These can aid in identifying optimum treatment for patients following surgery for colorectal cancer.

**Methods:**

Four focus groups were held with 29 participants from a multidisciplinary panel of healthcare professionals from two different NHS hospital sites in the UK. The discussions were audio recorded and underwent professional transcription. Transcripts were checked against recordings before undergoing thematic analysis using a realist approach.

**Results:**

Four themes were identified. The key barriers to standardised post-operative anaemia correction were a lack of protocoled guidelines or a defined pathway, insufficient education and training, and systemic barriers, such as financial drivers and drug availability. The key facilitator identified was collaboration and communication.

**Discussion:**

This study has identified several key barriers and thresholds which can be used in future studies to improve the standardised management of post-operative anaemia.

**Supplementary Information:**

The online version contains supplementary material available at 10.1007/s00384-024-04794-6.

## Introduction

Peri-operative anaemia is common in patients with colorectal cancer (CRC). Prevalence at time of diagnosis is up to 40% and most frequently attributed to iron deficiency [1, 2]. Iron deficiency anaemia is a common manifestation of CRC and a crucial criterion in identifying patients for urgent referral from primary care to colorectal surgeons to investigate potential cancers [3, 4]. It is linked to disease progression and cancer stage, resulting from blood loss commonly associated with intra-luminal tumours [5, 6]. Anaemia is an independent risk factor for adverse post-operative outcomes including complications, mortality and length of hospital stay (LOS) following colorectal cancer surgery [7, 8], However, allogeneic blood transfusion has been linked with adverse patient outcomes, higher mortality and increased cancer recurrence [9–11].

Peri-operative anaemia research has predominantly focused on the correction of pre-operative anaemia. Meta-analysis has highlighted an association between pre-operative blood transfusion with worse clinical (post-operative infection rates, LOS, mortality) and oncological outcomes (cancer recurrence) [10]. The use of pre-operative iron supplementation may reduce the need for blood transfusions and lead to shorter LOS, though longer term oncological and patient reported outcomes remain unclear [10]. This has therefore led to the development of patient blood management protocols to improve patient outcomes. However, randomised evidence has not demonstrated a similar benefit and meta-analysis has highlighted a paucity of high-quality evidence [12].

Post-operative anaemia (POA) has been researched to a lesser extent. Despite retrospective non-blinded studies leading to high-level randomised controlled trials in the pre-operative stage, this has not been replicated post-operatively. POA has been associated with worse clinical outcomes, increased LOS and lower survival rates [13, 14]. Effects of the treatment of anaemia post-operatively remain uncertain, with the impact on patient recovery and long-term outcomes currently unclear. Guidelines exist for the treatment of POA; however, these had ill-defined methodologies which were not specific in the context of CRC, lacked the clarity required to guide clinical practice and highlighted several areas where further research is required [15].

We aimed to understand current practice for the identification, correction and standardised care of POA in patients who had undergone surgical resection for CRC. Using focus groups (FG), we intended to investigate the facilitators of and barriers against standardised correction of post-operative anaemia and to define the haemoglobin (Hb) threshold to trigger correction of anaemia in this cohort of patients.

## Methodology

This qualitative research aims to capture barriers to and facilitators of the management of standardised POA in the context of surgery for CRC and is reported using the SRQR guidelines [16]. Ethical approval was acquired (IRAS 22/PR/1561).

### Focus Groups

Four FG were conducted. Purposive sampling of 29 participants from different multidisciplinary healthcare backgrounds took place to provide a rich perspective of those healthcare professionals (HCP) involved in the management of POA. An independent facilitator conducted these at two different NHS hospital sites. Staff were recruited to the study by email invitation circulated to all relevant staff groups (junior doctors, consultant anaesthetists, consultant surgeons, nurses, pharmacist and Transfusion Anaemia Lead) in each organisation by administrative staff on behalf of the research team. Emails included a copy of the participant information sheet and consent form. Written informed consent was obtained from each participant. FG were recorded using an encrypted digital audio recording device. Audio files were transcribed verbatim to text using a professional transcription service, before being checked for accuracy against recordings. Written notes were collected to identify key perceptions from each participant in relation to the main barriers and facilitators to the correction of anaemia in CRC patients.

### Data analysis

Qualitative data software (NVivo) was used. Transcripts were analysed against “programme theory” using realist evaluation to understand how treatment currently works in practice and what the challenges are. The complexity and variability in the management of peri-operative anaemia lends itself particularly well to realist study. Analysis involved iterative cycles of close reading, identifying points of interest in the transcripts and sharing the developing ideas with subsequent interview participants to help with theory refinement. Written notes collected during FG were coded and organised into categories to produce infographics of the overall results.

## Results

A total of 29 participants took part in the study across four FG and represented a wide range of HCP involved in the care and treatment of CRC patients undergoing surgery. The participant demographics can be seen in Table 1. The numbers within each FG comprised varied between four and eight participants.

**Table 1:** Demographic overview of participants

No	Focus group	Role	Gender	Time in role
1	FG1	Consultant Anaesthetist	F	5 yrs
2	FG1	Consultant Surgeon	F	12 yrs
3	FG1	Surgical Registrar	M	10 yrs
4	FG1	Clinical Nurse Manager	F	1 yr
5	FG1	Ward Sister	F	5yrs
6	FG1	FY1	F	1 yr
7	FG1	Consultant Surgeon	M	1 month
8	FG1	Senior House Officer	M	6 months
9	FG2	Consultant Anaesthetist	F	25 yrs
10	FG2	Consultant Surgeon	M	8 yrs
11	FG2	Senior Pharmacist	M	5 yrs
12	FG2	Transfusion Anaemia Lead	F	10 yrs
13	FG2	Clinical Nurse Specialist	F	6 yrs
14	FG2	Ward Sister	F	1 yr
15	FG2	Senior Clinical Fellow	M	3.5 yrs
16	FG2	Ward Sister	F	1 yr
17	FG2	Senior Sister	F	4 yrs
18	FG3	CT2	F	8 months
19	FG3	FY2	F	8 months
20	FG3	FY1	F	8 months
21	FG3	Locum Surgeon	M	18 months
22	FG4	Senior Colorectal Nurse	F	4 yrs
23	FG4	Consultant Anaesthetist	F	4 yrs
24	FG4	ST3 Registrar	M	6 months
25	FG4	Medical Student	M	5 yrs
26	FG4	FY1	F	8 months
27	FG4	Ward Sister	F	5 yrs
28	FG4	Consultant Surgeon	M	10 months
29	FG4	Clinical Nurse Specialist	F	3 yrs

Thematic analysis of the FG transcripts identified the facilitators and barriers to the standardised management of POA and an agreed Hb threshold for treatment.

The themes identified as barriers were:

1. Lack of protocoled guidelines and defined pathway
2. Education and training
3. Systemic barriers

The facilitators were identified as:

1. Collaboration and communication

**Fig. 1:**
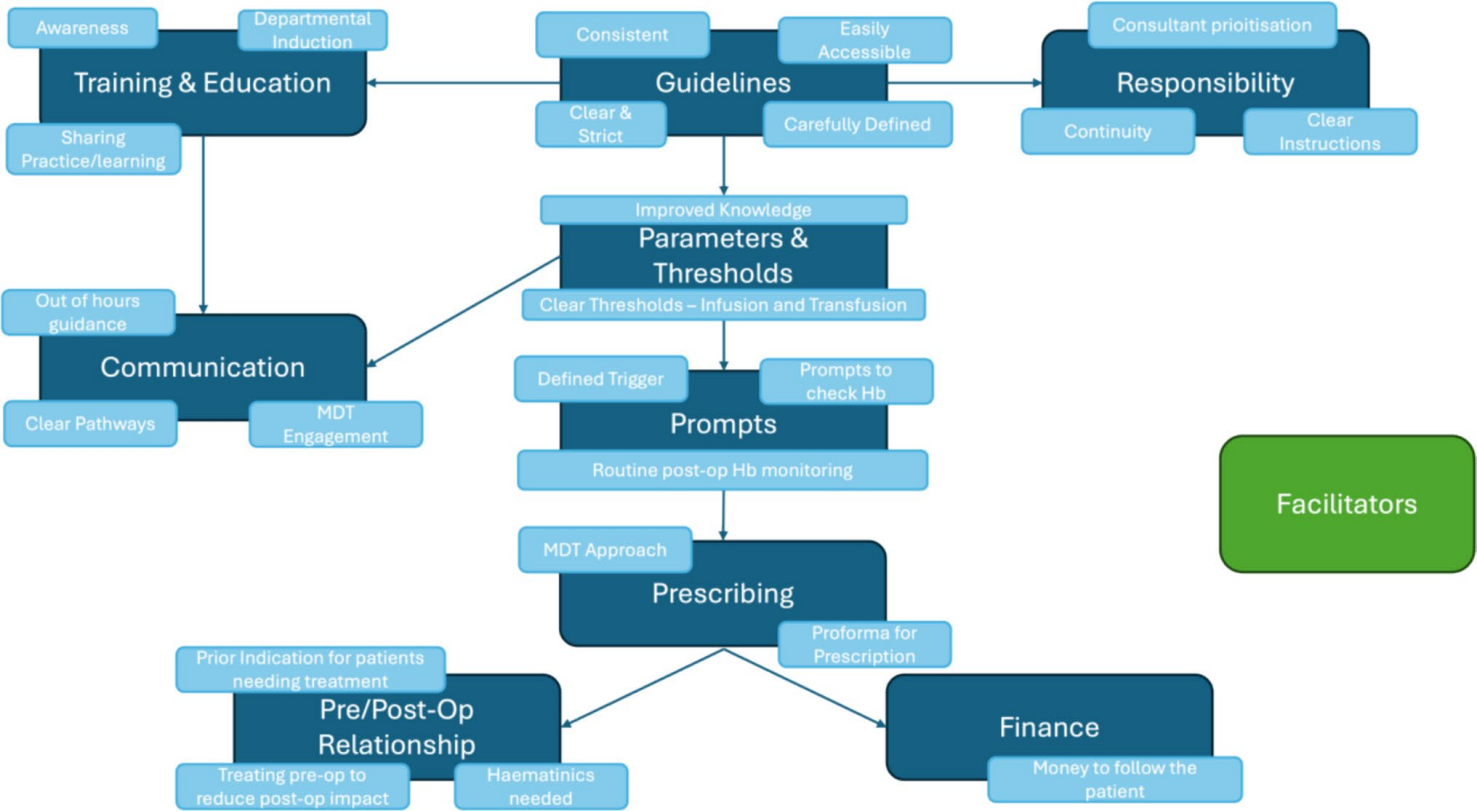
Infographics of a summary of facilitators to the correction of anaemia

**Fig. 2:**
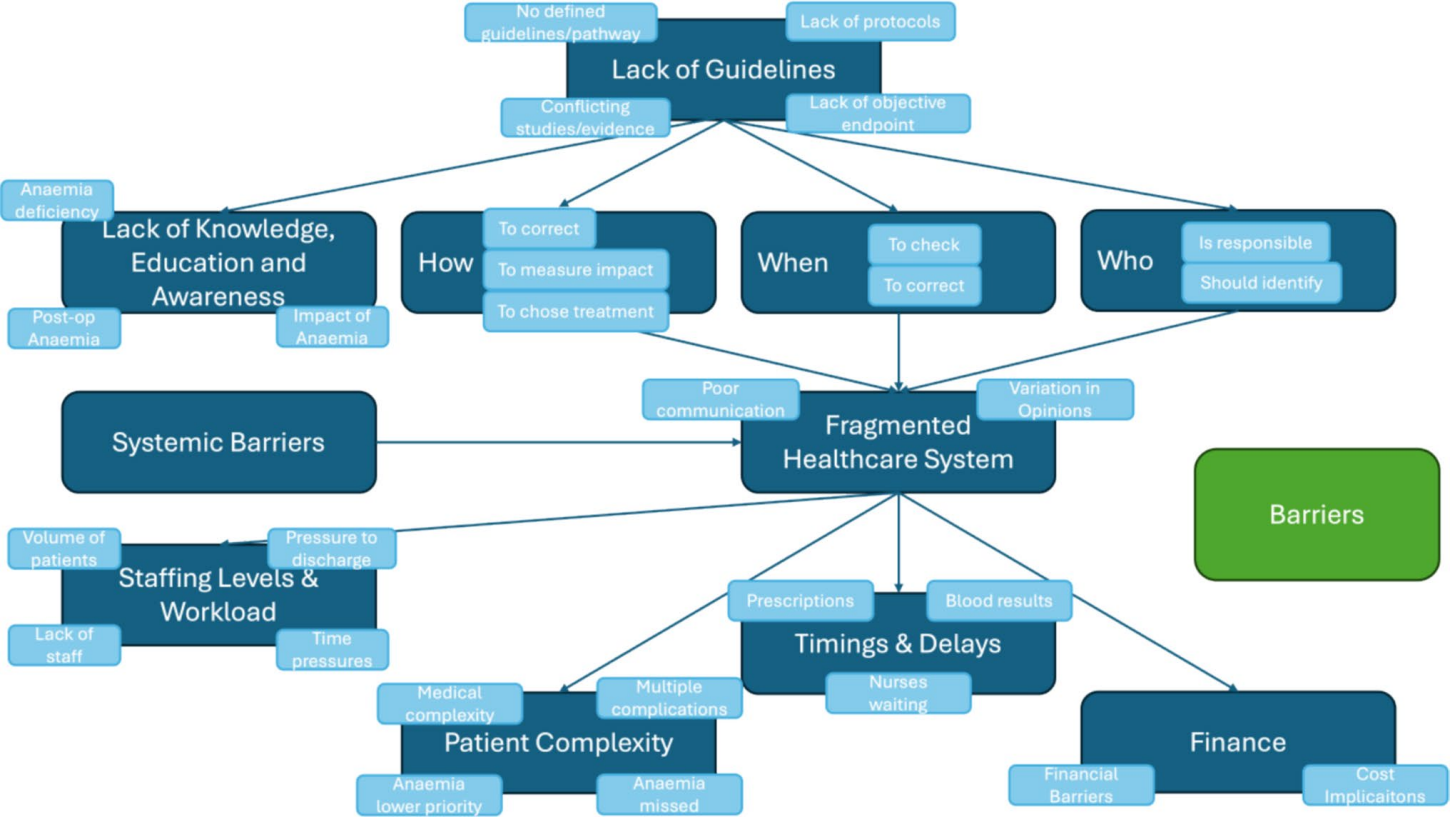
Infographics of a summary of perceived barriers to the correction of anaemia

The written notes were collated to reflect these and can be seen in Figs. 1 and 2. The four themes are explored below, with further quotes available in Appendix 1.

### Lack of protocoled guidelines and defined pathways

There was a lack of clarity on how PAO should be managed, with regards to patient identification, treatment decisions and with whom the responsibility for this lies.

As a Transfusion Anaemia Lead commented:

I think that what we are lacking is a protocol and clear guidance of which day and when we are going to [check and correct anaemia] (FG2).

There was a general consensus within the groups regarding lower thresholds for treatment; however, there was a lack of granularity when this was applied to individual cases with variation in comorbidities and other factors affecting outcomes:

Numbers are fine, but you could have a patient with Hb of 70 and they are not symptomatic, whereas you might also have a patient who has Hb of 95 and is struggling because of comorbidities and you want to be able to give them the best possible chance (FG4).

There was agreement within the groups that those with symptomatic anaemia should be treated/transfused irrespective of their underlying Hb.

the threshold can be reduced or sort of increased if the patient is symptomatic. If they’re breathless and then they have a Hb of 90 or 100, then you would transfuse them (FG4).

Uncertainty mainly surrounded the treatment of those with POA, above the agreed threshold for treatment with blood transfusion and who remained asymptomatic, some of whom may still have had a significant drop in their haemoglobin level.

if they’ve gone from 120 to, you know, 80 or 90, that’s quite a big drop and they’re more likely to decompensate from that (FG3).

it’s a little bit more left to each one’s judgement…..these patients could still fall within the anaemic range, for example could have a Hb of between 90–110 (FG4).

Although there was agreement over who definitely needed a post-operative blood transfusion, there was a lack of clarity regarding iron infusion.

I don’t know what the actual specific guidelines are giving Monofer [brand of IV Iron Infusion], so, I’m not sure (FG3).

if it was a full blood transfusion, you were saying, would be 70, but if you’re using IV [iron] Fusion or oral tablets, it’s different (FG1).

I feel like the guidance isn’t that clear with Monofer infusions ….when you repeat them………. I’m not 100% sure (FG3).

Several junior doctors (*n* = 4) observed that lack of clarity was sometimes compounded by senior staff who had differing practice preferences in relation to administering IV iron infusions.

certain consultants quite like Monofer, and certain consultants can’t be bothered with it, basically.…. I always wondered was there actually strict guidance? (FG3).

### Education and training

This theme covered both education of doctors and patients. Junior doctors felt as though their knowledge and understanding of POA and its treatment could be improved. However, they found it difficult gaining this knowledge as different consultants wanted to do different things and therefore there was a lack of consistency with practice and education.

it’s like a guessing game based on your clinical knowledge, which you don't trust, because you’re so junior (FG1).

Consultants felt that short rotational training had significantly impacted on junior doctors, with less embedded time within individual specialities making it difficult for them to familiarise themselves with and recall the relevant protocols.

our junior doctors rotate so frequently, don’t they? (FG2).

Patient education was also discussed. Educating and enabling patients would allow them to approach GPs for treatment and allow for continued treatment of peri-operative anaemia in the community.

we have to enable the patient. We have to give them the information. We have to educate them. We have to say, it’s your responsibility. You have been anaemic. You have been iron deficient, you need to have these tools and this knowledge to be able to go to your GP or wherever and ask for help or ask for investigations, ask for treatment (FG2).

### Systemic barriers

The financial impact of blood transfusion was well understood by the participants of the FG and played a role in treatment decision-making.

…..we’ve been developing these figures and it’s very interesting because if we give an iron infusion rather than a blood transfusion, we save the Trust over £1,000……so, when we treat patients with IV iron, we’re actually saving, and we worked out, we saved the Trust alone in a year about six and a half million pounds (FG2).

The choice of drugs as an inpatient favoured one brand over another due to the cost of the drug:

CosmoFer [brand of IV iron infusion], ………… it costs a lot less [than MonoFer], which is why it wanted to be the drug of choice (FG4).

Discussions took place concerning use of oral iron as an outpatient rather than keeping patients in hospital to treat their iron deficiency.

And they [oral iron replacement] probably cost effective, I thought it would be a reasonable place to start (FG1).

The other systemic barrier identified was the primary and secondary care interface. The difficulties associated with managing patients between the two was highlighted.

discharging patients with anaemia was a big issue (FG2).

GPs are so busy now like can you say please …. do bloods in six weeks (FG1).

There was a protocol from secondary care for the ongoing management and monitoring of peri-operative anaemia; however, members of the FG stated this often was not able to be completed in primary care.

the blood form for them to go to their GP ……If they can’t get hold of GP, it falls on us. So, they tend to come in here (FG2).

### Collaboration and communication

These were key facilitators of good management of POA. Effective lines of communication and a shared vision between different HCP roles improves continuity of practice throughout the patient’s peri-operative journey. One of the nurses described the teamwork.

that is dependent on the advice given by – sort of, at discharge by the doctors (FG4).

A collaborative piece of work from one of the hospital sites led to an info-graphic that ensured patients were identified and treated for anaemia early in their treatment pathway (Appendix 2).

…the sooner we start the better, because it just helps that patient to have more energy to be able to do the things that they need to do to recover (FG4).

The impact of treating peri-operative anaemia early and the positive impact this has on patients was then discussed.

I really believe that a better preparation before the surgery will have a direct impact on not having anaemia after the surgery (FG2).

If we can optimise people while they’re here, before they go home, that is always the best thing. (FG4).

#### Thresholds

There was good consensus regarding the Hb thresholds for treatment of POA within the FG. The Hb range for consideration of treatment with iron was 70–130 g/L. Those with an Hb above 130 g/L would not be considered anaemic. Those with an Hb lower than 70 g/L, or 80 g/L with concurrent cardiac issue or ischaemic heart disease, were deemed to have anaemia requiring treatment with transfusion of packed red cells.

…there are relatively firm guidelines on when to transfuse (FG3).

a patient with a Hb of 70 would require a good background of vascularity and oxygenation for optimal healing and recovery following a resection involving anastomosis, which would have incurred acute bleeding during the operation and that therefore a blood transfusion was required (FG3).

….we know that transfusion itself can bring risks for the patients who are diagnosed with cancer as it has got long-term negative effects and we would like, by all means, to avoid it (FG4).

## Discussion

POA is a complex issue. The management of pre-operative anaemia is well researched; however, similar time and effort has not been invested to establish best practice in the management of POA, especially in those patients who have undergone resectional surgery for CRC. These FG have identified three key barriers and one facilitator for the standardised treatment of POA. A lack of protocoled guidelines and a defined pathway, insufficient education and training of different multidisciplinary team members and systemic issues formed barriers whilst collaboration and communication among the team was identified as a key facilitator. The FG also identified a clear threshold for consideration of treatment of POA with iron replacement as an Hb 70–130 g/L, providing there was no concurrent cardiac disease where a higher Hb threshold for blood transfusion should be considered.

A lack of clear guidelines for CRC patients exists despite the publication of the international consensus statement on the management of POA [15]. The FG required clear contextual guidance on specific details related to diagnosis, blood tests including haematinic studies, treatment options and follow-up requirements. A lack of consistent and protocoled guidelines for management of peri-operative anaemia has been identified elsewhere by clinicians who discussed the “unwarranted variation” in diagnosis and management [17]. Current published literature is limited in respect of POA and therefore makes drawing conclusions in this area challenging at present. Concerns around education and training for junior doctors has been raised in other focus group-based studies, with high-frequency clinical rotations leading to a “nomadic” nature of training [18]. The potential facilitator identified in this study, being the need for collaboration and communication has been clearly identified elsewhere, with improvements leading to effective organisational and clinical management of anaemia [19].

This was a high-quality qualitative study. The main strength was the independence of the FG facilitator, who had significant experience in qualitative evaluation in healthcare, and the data coder, ensuring impartial and reliable qualitative data. High-quality purposive sampling was reflected in the range of HCP backgrounds in the FG. Additionally, the utilisation of realist evaluation allowed for open interpretation and aided free flowing discussion and the development of ideas amongst HCP participants. However, participants were invited to take part in the study from only two NHS Trust hospitals in the South West of England and results could therefore not be representative of the overall views of peri-operative anaemia management across the UK. With different levels of seniority within the FG reflecting the purposive sampling, conformity bias due to varying seniority is a concern.

## Conclusion

This study brings focus to the less researched topic of POA, highlighting four key themes, which represent barriers and facilitators to its effective and standardised management. It has also highlighted the areas of future research to achieve consensuses and guidance in developing and testing a standardised protocol for the management of POA in patients undergoing surgery for CRC.

## Supplementary Information

Below is the link to the electronic supplementary material.

Supplementary file1 (DOCX 23 KB)

Supplementary file2 (DOCX 129 KB)

## Data Availability

No datasets were generated or analysed during the current study.
