# Supplementary material for: Management of post-operative anaemia in patients undergoing surgery for colorectal cancer: a qualitative focus group-based study
Source: Int J Colorectal Dis. 2025 Jan 15;40(1):14. doi: 10.1007/s00384-024-04794-6 (PMC11735509; doi:10.1007/s00384-024-04794-6)
Supplement: Supplementary file 1 — Supplementary file1 (DOCX 23 KB) [file 384_2024_4794_MOESM1_ESM.docx]

Appendix 1

**THEME 1: Lack of protocolised guidelines and defined pathway**

- 1. ***Mid-range Hb levels***

Transfusion Anaemia Lead:

*“I think that what we are lacking is a protocol and clear guidance of which day and when we are going to* [check and correct anaemia]*”*

Consultant Surgeon:

*“…the issue is more in a stable patient without symptoms, that we’re about to send home and they’re anaemic. So, what to do with those patients?...it’s a little bit more left to each one’s judgement…..these patients could still fall within the anaemic range, for example could have a Hb of between 90-110, and yet be discharged”*

Locum:

*“So, even if they're not necessarily below the 70 threshold, if they've gone from 120 to, you know, 80 or 90, that's quite a big drop and they're more likely to decompensate from that. So, you're probably more likely to transfuse”*

FY1:

*“….if somebody's been chronically anaemic pre-operatively, and then they persistently are post-operatively, you know, their functioning states, their body's kind of adapted to the fact that they're running anaemic. So, say somebody's Hb is like a 100 pre-op, and then 90 post-op, you'd be like, oh, that's actually not actually that bad in terms of…. it's not that different to baseline. But if somebody's gone from a normal haemoglobin of 130 and then suddenly dropped to 90, I'd be far more concerned about, firstly, the quantity of bleeding, but also their kind of mechanisms of how their body is going to adapt to that. And they're more likely to be sort of acutely symptomatic from that, compared to somebody that was probably a bit anaemic for operation anyway”*

ST3 Registrar:

*….and the threshold can be reduced or sort of increased if the patient is symptomatic. If they’re breathless and then they have a HB of 90 or 100, then you would transfuse them.”*

5^th^ year Medical student:

*“…from my medical perspective, we’re told the whole 70 threshold in the textbook, but the experience of seeing….in real life, it tends to be sort of more case-by-case discussion…. but also that you’ve got the threshold there as well as sort of backup, use as evidence for any transfusions, iron related, or blood products as well”*

Clinical Nurse Specialist:

*“Numbers are fine, but you could have a patient with Hb of 70 and they are not symptomatic, whereas you might also have a patient who has Hb of 95 and is struggling because of comorbidities and you want to be able to give them the best possible chance to become ambulant and recover from surgery”*

- 1. ***Lack of clarity in the use of IV iron infusions***

FY1:

*“I think we tend to give them a shot of Monofer before they go home. If there are iron deficient, you would hope with a view to, you know, replenishing their stores, and then as they recover postoperatively, you'd hope they wouldn't then become anaemic again. If you're assuming that their pre-existing anaemia was probably related to their colorectal cancer, and then with the second hit of them having an operation, I don't know. Again, I don't know what the actual specific guidelines are giving Monofer, so, I'm not sure but in my head, that would be how I would understand it.”*

Junior doctor:

*“….if Monofer used post op no further top up, ie from oral, would be needed…...so, if you gave them Monofer, so, IV iron to top up their iron stores, they, in theory, wouldn't need any follow-up supplementation because you've reversed their cause of the iron deficiency by then. So, I think maybe if you top them up prior to discharge, you would hope that they won't go on to need any further supplementation, I think”*

FY1:

*“….certain consultants quite like Monofer, and certain consultants can't be bothered with it, basically. …..so, like sometimes it would be…. we’re definitely giving this patient Monofer and then if there's another consultant on, they wouldn't even consider it. …. I always wondered was there actually strict guidance?”*

CT2:

*“I feel like the guidance isn't that clear with Monofer infusions ….when you repeat them, and I think from what I've been told in the past, it's like three months, every three months if their iron levels are still low, but I'm not 100% sure”*

Locum:

*“….. if their operation’s finished and, you know, that if they're stable and their haemoglobin is not worryingly low, like you're not worried about, you know, needing to give them blood….. is there really any benefit in trying to pump them full of iron”*

**THEME 2: EDUCATION AND TRAINING**

**2.1 Junior doctors**

FY1 doctor:

*“So, we’re quite nervous like you don’t want to give it and then make the surgeon angry, and get in trouble, or anything, you know…. and otherwise, it's like a guessing game based on your clinical knowledge, which you don't trust, because you’re so junior”*

**2.2 Patient education**

Transfusion lead:

*“……..people say to me: Why has it only just come to light? Why have I been signed off work for the last 3 yrs? Why wasn’t it identified earlier? Why am I so poorly? And sometimes, it’s just because they have surgery that it’s been discovered, in fact, quite often, so it’s huge but we’re a bit stuck”*

*“So, really my message is that we have to enable the patient. We have to give them the information. We have to educate them. We have to say, it’s your responsibility. You have been anaemic. You have been iron-deficient, you need to have these tools and this knowledge to be able to go to your GP or wherever and ask for help or ask for investigations, ask for treatment”*

**THEME 3: COLLABORATION AND COMMUNICATION**

Clinical Nurse Specialist:

*“…the sooner we start the better, because it just helps that patient to have more energy to be able to do the things that they need to do to recover”*

Ward Sister:

*“I really believe that a better preparation before the surgery will have a direct impact on not having anaemia after the surgery”*

Senior Colorectal Nurse:

*“…it is best to optimise patients before they are discharged ……once they do get home, particularly if they’ve had major surgery, coming back into the hospital a week after discharge is not necessarily the easiest thing for people to be able to do. If we can optimise people while they’re here, before they go home, that is always the best thing.”*

Clinical Nurse Specialist:

*“…..when we’ve reviewed a patient, we’ll always give them a form and when we treated them, ask them to get their bloods checked at six weeks post-infusion. And generally, follow that up with -- we can follow up with a second iron infusion if needed. We obviously always try and maximise the dose, so we’ll give them…… optimise their Hb with iron rather than blood….* *obviously, that is dependent on the advice given by -- sort of, at discharge by the doctors…..but if they aren’t given any specific advice about blood monitoring, then at that point they’ve always got a blood form to get their bloods checked”*

Ward Sister:

*“….probably on TrakCare………TrakCare is where we -- we’re looking at the TrakCare notes all the time every day……and that’s where the nursing staff update continuously. So, if we see it there, then we'll be reminding the doctors. Should we have given this? Have we given it? That is probably the best place for…… where the bed space is. Yeah, we can update it. Well, the doctors can update that bit as well. Everybody has access to that bit……”*

CNS:

*“You can get alerts, well, can’t you? You can get an alert put on TrakCare. So we’ve -- I know [name] has set up for the alert now so that patients who are known to the anaemia service, if they’re admitted to the hospital, it flags an alert on TrakCare. So, whether you could put an alert on there to say that this patient requires IV treatment for an iron deficiency before they’re – before discharge”*

**THEME 4: SYSTEMIC BARRIERS**

***4.1 Financial/business model***

A Transfusion Anaemia Lead explained:

*“…..we’ve been developing these figures and it’s very interesting because if we give an iron infusion rather than a blood transfusion, we save the Trust over £1,000……so, when we treat patients with IV iron, we’re actually saving, and we worked out, we saved the Trust alone in a year about six and a half million pounds”*

*“….it needs to be recognised by the people who hold the purse strings, the IV iron, intravenous iron should be used and should be used more than it is at the moment. We’re just cherry picking at the moment the people we’re treating”*

A Senior Pharmacist went on to explain:

*“...if you save on your blood transfusion, that’s a completely different budget….the problem we have, is that pharmacy budget is still ring fenced to a certain extent”*

Transfusion Anaemia Lead:

*“…. because if we give it as a day case, we have an income from that, but if it’s given on the wards, we don’t have an income from that. So, there's a great resistance to giving IV iron, from the financial point of view”*

A Senior Pharmacist:

*“there's a big caveat in terms of the IV iron in that this Trust* [Yeovil] *is actually quite a high user compared with others [e.g. Exeter] in the region, which some of the budget holders are quite concerned about”*

Clinical Nurse Specialist:

*“CosmoFer, which is a lot more – it’s a lot more challenging.….. I think it’s given over a more lengthy period of time. It’s – it costs a lot less, which is why it wanted to be the drug of choice. But I think needs to be administered over about five hours, it involves more monitoring from a nursing point of view at ward level”* (FG4)

***4.2 Primary Care/Secondary Care interface***

Transfusion Anaemia Lead:

*“…[we] have to rely on primary care services to monitor and address any ongoing problems…… you know, and I think the problem is if we don’t have the plan for caring for them after discharge, they're just going to be readmitted”*

Clinical Nurse Specialist:

*“…. at the time of discharge, we give our follow up call and the blood form for them to go to their GP and get their bloods repeated either in four weeks’ time or six weeks’ time……If they can’t get hold of GP, it falls on us. So, they tend to come in here. We’d meet them at phlebotomy, give them a blood form and get their bloods done and then follow up again, so we don’t lose them”*

Clinical Nurse Specialist:

*“Mostly, colorectal patients, they're not discharged on oral iron because they have urgency or frequency of stools post-surgery so we tend to bring them back and give IV iron, not oral……the same with patients who’ve had an anastomosis – just not appropriate”*

Locum:

*“I don't think iron, you know, oral supplements are used as that frequently, particularly in a ward setting, and often find that, you know, most patients, even if they have a Monofer infusion as an inpatient, they won't go home with oral supplementation to follow that up. It's often just a one-off infusion and then you sort of”*

Transfusion Anaemia Lead:

*“….. we could make such difference to the workload of the NHS if we treated people who are iron deficient when it, when their symptoms first appeared so going out into the neighbourhood, going out into primary care and identifying those people and treating them. And some of the time, it would require intravenous iron. Some of the time, it would be proper guidance on oral iron which is not an easy drug take”*
