# Supplementary figures and images for: Management of post-operative anaemia in patients undergoing surgery for colorectal cancer: a qualitative focus group-based study
Source: Int J Colorectal Dis. 2025 Jan 15;40(1):14. doi: 10.1007/s00384-024-04794-6 (PMC11735509; doi:10.1007/s00384-024-04794-6)

Supplementary Appendix 1 – Infographic for early detection of anaemia


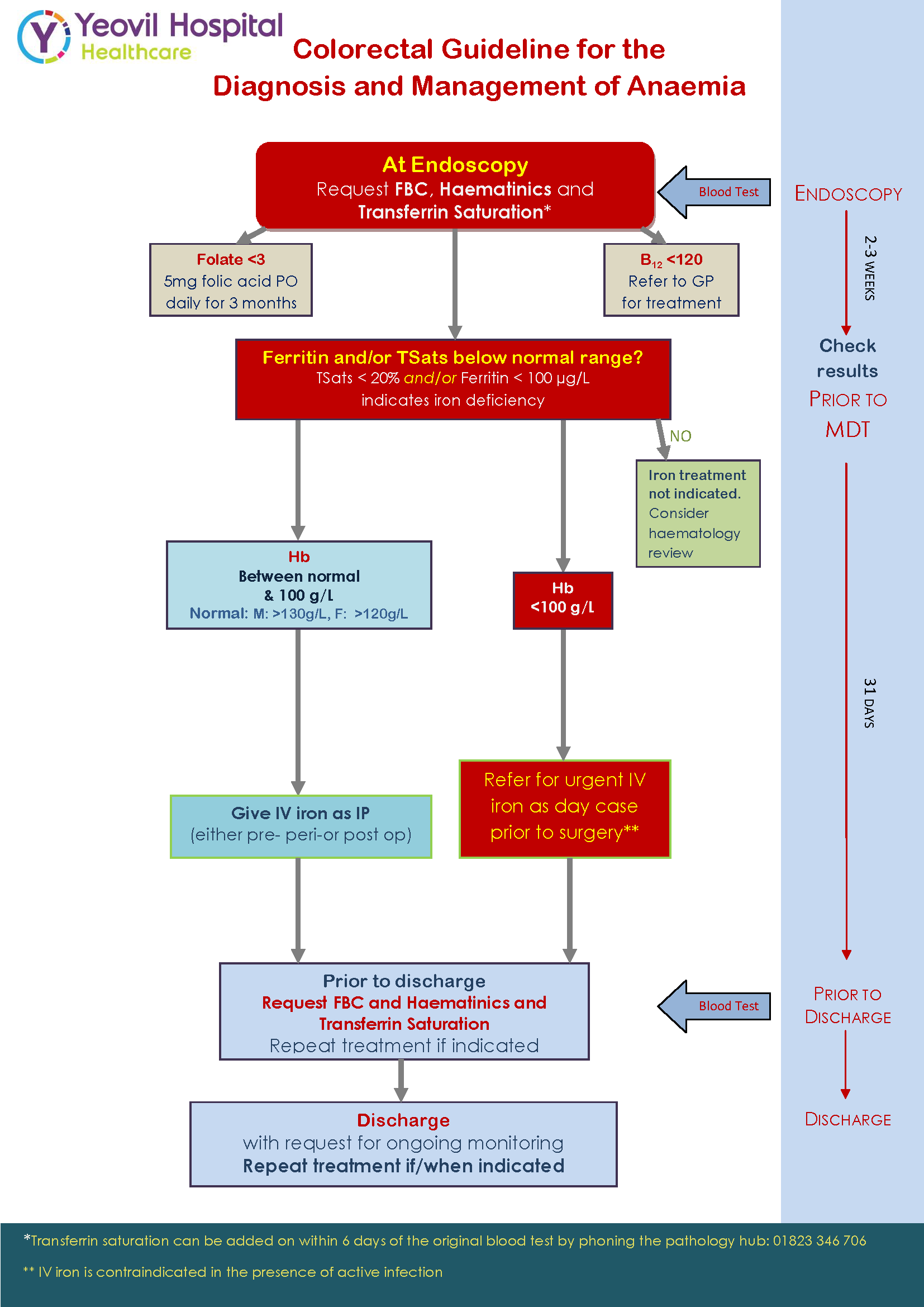

Supplement: Supplementary file 2 — Supplementary file2 (DOCX 129 KB) [file 384_2024_4794_MOESM2_ESM.docx]
